# Ultrasound and magnetic resonance imaging are not interchangeable to assess the Achilles tendon cross-sectional-area

**DOI:** 10.1007/s00421-016-3500-1

**Published:** 2016-11-12

**Authors:** Annika Kruse, Savvas Stafilidis, Markus Tilp

**Affiliations:** 1 Institute of Sports Science, University of Graz, Mozartgasse 14, 8010 Graz, Austria; 2 Centre of Sport Science, University of Vienna, Auf der Schmelz 6a, 1150 Vienna, Austria

**Keywords:** Magnetic resonance imaging, Ultrasonography, Achilles tendon, Reproducibility, Cross-sectional area

## Abstract

**Purpose:**

The major aim of this study was to compare ultrasound (US) and magnetic resonance imaging (MRI) measurements of the Achilles tendon cross-sectional area (CSA). Further aims were to conduct reliability analyses and to assess the influence of transducer pressure on the tendon properties in US measurements.

**Methods:**

The Achilles tendon CSA of 15 participants was assessed at two positions with US and MRI by use of a standardized protocol. Method comparison was performed by two-way analysis of variance (ANOVA) and paired *t* test. Reliability was assessed by coefficients of variation (CV), intraclass correlation (ICC_2,2_), standard error of measurement (SEM), and minimal detectable change (MDC_95_). A paired *t* test was performed to investigate the effect of probe pressure on tendon CSA and thickness.

**Results:**

Mean US measurements provided a ~5.5% smaller CSA compared to MRI measurements. Intra-rater reliability analyses of US demonstrated CV values of 1.5–4.9%, ICC of 0.89–0.97, SEM and MDC_95_ values of 0.22–0.77 mm^2^ and 0.61–2.16 mm^2^ for both raters, whereby CV values for intra-rater reliability of MRI ranged from 1.0 to 3.7%. Inter-rater reliability was lower for both modalities. Pressure applied on the transducer altered Achilles tendon CSA and thickness significantly (*p* < 0.05).

**Conclusions:**

Our findings show that US and MRI cannot be used interchangeably for Achilles tendon CSA assessments, however, each imaging modality separately is reliable to assess this property. Pressure applied on the transducer during US measurements causes alterations of the tendon’s morphology and should be avoided.

## Introduction

Ultrasound (US) and magnetic resonance imaging (MRI) are the most frequently used imaging methods to assess the cross-sectional area (CSA) of tendons (Pierre-Jerome et al. 2010). Both methods are well-established non-invasive diagnostic tools to evaluate the Achilles tendon (AT) mechanical properties in biomechanical research, whereby MRI has often been the preferred imaging modality (Jacobson 2005; Rasmusson 2000). Nevertheless, precision and measurement reliability of US and MRI are essential (Skou and Aalkjaer 2013) when the morphological and mechanical properties of tissue are assessed, e.g., to investigate intervention-related alterations. In this context, several studies examined the reliability of either US (Brushoj et al. 2006; Dudley-Javoroski et al. 2010; Foure et al. 2011; Intziegianni et al. 2015; Kubo et al. 2014; Milgrom et al. 2014; Ying et al. 2003) or MRI (Arampatzis et al. 2010; Brushoj et al. 2006; Hansen et al. 2003; Kubo et al. 2002; Magnusson et al. 2001) measurements of the AT CSA and demonstrated predominantly good to excellent reliability for US applications. In contrast, investigations conducted with MRI showed varying results regarding the reliability (Brushoj et al. 2006; Hansen et al. 2003; Kubo et al. 2002; Magnusson et al. 2001).

To ensure a high reliability and to enhance image quality, especially in US examinations, several aspects have to be considered (e.g., positioning of the subject, additional markers and devices (Foure et al. 2011; Ying et al. 2003), joint fixation, probe alignment, and probe pressure (Brushoj et al. 2006; Dudley-Javoroski et al. 2010; Milgrom et al. 2014). Furthermore, it is crucial to ensure the same measurement position during examinations to account for the variability of the CSA throughout its length (Arampatzis et al. 2010; Kongsgaard et al. 2005; Magnusson and Kjaer 2003). In this context, a lack of joint fixation (Arampatzis et al. 2006) might lead to different results due to joint rotations. Referring to the aforementioned considerations, the application of US for the investigation of the AT CSA should be justified by its comparability or interchangeability with the well-established imaging method MRI (Jacobson 2005; Rasmusson 2000).

To the best of our knowledge, only Brushoj et al. (2006) and Bohm et al. (2016) examined US and MRI findings of the AT CSA. In addition, the effect of transducer pressure on the AT CSA has not been investigated yet.

Therefore, the major aim of this study was to evaluate and compare the interchangeability as well as the reliability of US and MRI measurements of the AT CSA using a standardized examination protocol. Furthermore, the study aimed to investigate the influence of transducer pressure on the AT. We hypothesized that US and MRI findings are reliable and comparable when a standardized examination protocol is used. Moreover, we expected that transducer pressure will alter the morphological properties of the AT.

## Materials and methods

### Subjects

The sample size was determined by a power calculation (G*Power, Faul et al. 2007) based on data published by Brushoj et al. (2006): Our calculation was based on 7% (5% SD) Achilles tendon CSA difference between MRI and US measurements and resulted in a required inclusion of 12 subjects to receive a power value of 0.90 (large effect). To account for any possible dropout, 15 healthy subjects (Table 1) were included in this study. All measurements were made on the right leg of the participants. No one reported any history of AT injury and informed consent was obtained from all individual participants included in the study. The study was approved by the Ethics Committee of the University of Graz, Austria.

**Table 1:** Mean (±SD) of the subject’s characteristics

Sex	Number (*n*)	Age (years)	Body mass (kg)	Height (cm)	Side tested
m	11	31.8 ± 5.0	71.5 ± 6.0	178.2 ± 6.0	Right
f	4	22.8 ± 3.0	60.0 ± 2.2	164.0 ± 4.5	Right

*m* male, *f* female

### Measurements of the Achilles tendon cross-sectional area

#### US examinations

**Fig. 1:**
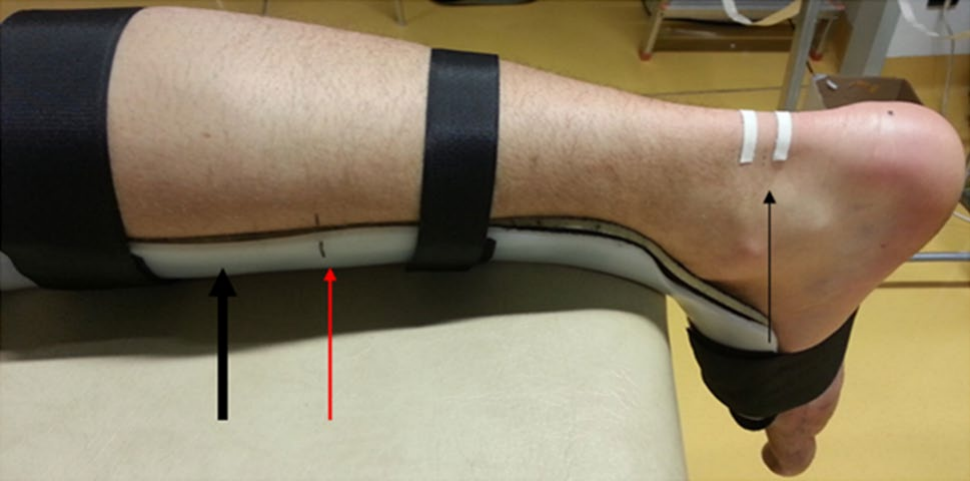
Subject’s right lower leg with the custom made splint (*black arrow* in *bold*). The *red arrow* indicates the alignment line drawn on the sleeve of the splint and the skin of the subject. The *thin black arrow* shows the adhesive tape used to locate the measurement positions. The *black spot* on the heel represents the tuberositas calcanei (color figure online)

US examinations were conducted at the Institute of Sports Science of the University of Graz. Subjects were scanned by two raters on two days. B-mode ultrasonography (MyLab60; Esaote S.p.A., Genova, Italy) was used to determine the AT CSA. All measurements were obtained with a 4- to 13-MHz linear-array transducer (LA 523; Esaote S.p.A., Genova, Italy; maximum depth 30 mm; focal zone 0.7–1.4; axial and lateral resolution 0.154 × 0.260 mm; no image filter). A stand-off gel pad (SONOKIT soft 200 × 100 × 20 mm; SONOGEL, Bad Camberg, Germany) was placed between the skin surface and the probe. Subjects lay prone on the examination table. The ankle joint (Fig. 1) was stabilized at ~90° with a custom-made splint (Ortho-Aktiv; Graz, Austria) and the ankle joint angle was controlled with a goniometer (Ka We V01, Medizintechnik). Moreover, to ensure identical positioning of the splint throughout the entire measurement sessions, an alignment line was drawn on the sleeve and the skin (Fig. 1).

**Fig. 2:**
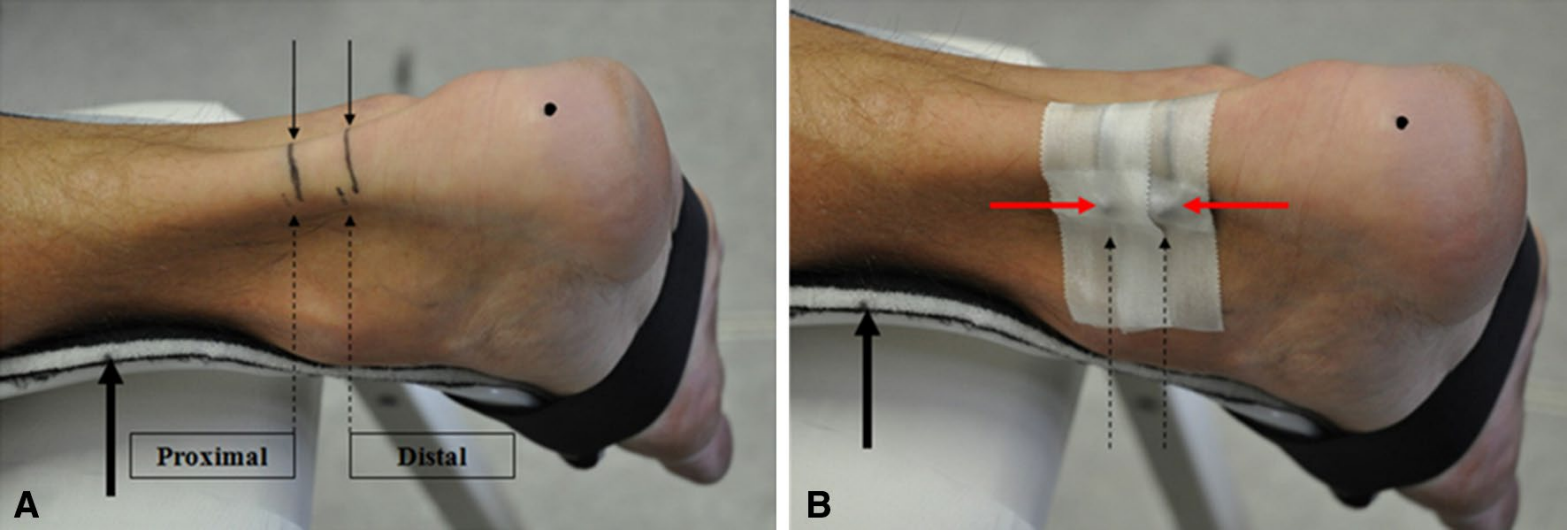
Subject’s ankle joint with the attached splint (*arrow* in *bold*) **a** initial preparation for measurements and **b** the ankle joint prepared for the MRI examination. *Black arrows* indicate measurement landmarks, whereby *dashed arrows* indicate the measurement positions (distal, proximal) defined with respect to the footprint of the transducer. *Red arrows* indicate the spherical markers attached to the measurement positions. The *black spot* on the heel indicates the tuberositas calcanei (color figure online)

Based on previous studies (Brushoj et al. 2006; Dudley-Javoroski et al. 2010; Foure et al. 2011; Intziegianni et al. 2015; Kallinen and Suominen 1994; Kongsgaard et al. 2005; Magnusson et al. 2001; Milgrom et al. 2014; Rosager et al. 2002; Waugh et al. 2012; Ying et al. 2003), we conducted the CSA measurements at the level between the malleoli and additionally at a second more proximal position (Fig. 1). The curved path from the anterior aspect of the tuberositas calcanei to the midpoint of the medial and lateral malleolus was measured and a solid line was drawn at this point (medio-lateral direction). Furthermore, a second solid line was drawn 15 mm proximally of the first one. In addition, owing to the width of the footprint of the transducer (surface area that is in contact with the skin; 50 × 8 mm), dashed lines were drawn 4 mm proximally of the first and the second solid line, respectively. These lines were defined as distal and proximal position, respectively (Fig. 2a).

Subjects were instructed not to remove the marks throughout the entire measurement sessions. An adhesive tape (width 3 mm) was fixed directly below the two solid lines. Due to its anechoic behavior, the tape was clearly visible as a shadow in the ultrasonic images, and therefore, it was used to define the lower boundary of the selected measurement positions (Fig. 1).

For reliability analysis, each rater randomly obtained three images in each measurement session (i.e., each day) at both measurement positions whereby different pressure was applied. During all measurements, the US probe was placed perpendicular to the AT and images were captured by removing and repositioning the probe between scans. Prior to the measurement sessions, the subjects did not perform any warm-up and the room temperature was kept constant at ~20.5 °C.

**Fig. 3:**
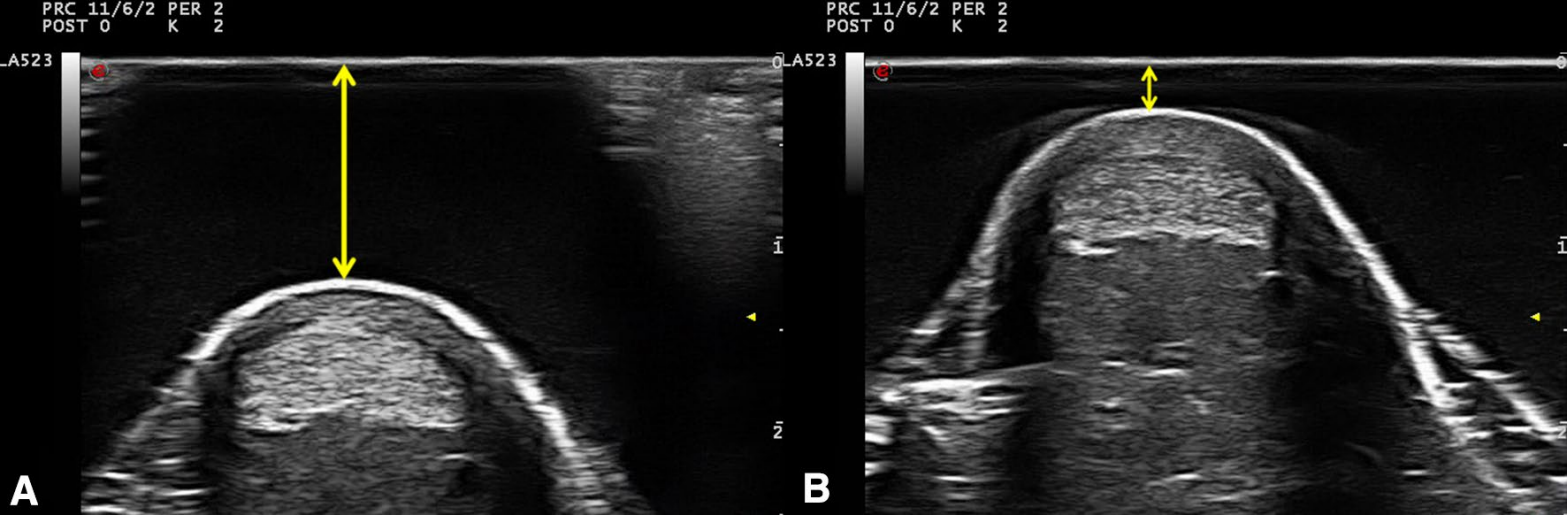
US images of the Achilles tendon CSA with **a** minimal and **b** maximal pressure application. The *yellow arrows* show the distance between the gel pad surface and the skin (color figure online)

Despite the use of a stand-off gel pad, we could not avoid applying pressure with the transducer to achieve clear US images of the Achilles tendon CSA. To estimate the effect of the applied pressure, we additionally obtained an ultrasonic video of the tendon CSA. We recorded image sequences of the subject’s CSA with minimal (MIN) and maximal (MAX) pressure of the transducer to the adjacent gel pad by steadily augmenting the applied pressure. Due to technical limitations, it was not possible to measure the mechanical compression characteristics of the gel pad, and therefore, solely the distance between the gel pad and the skin surface was used as index of the applied force (Fig. 3a, b). Altogether, the applied pressure on the tendon area was examined in 24 videos resulting from both measurement sessions.

**Fig. 4:**
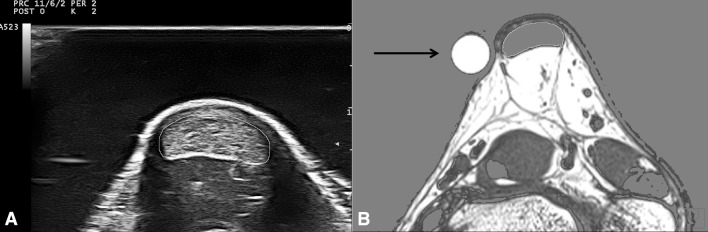
**a** Transversal US image demonstrating the manually outlined Achilles tendon CSA (*yellow* shape) and **b** transversal T1-weighted MRI image showing the automatically outlined Achilles tendon CSA (*yellow* shape) with the spherical marker (*black arrow*) to the left (color figure online)

All US images were analyzed with an open-source image processing program (ImageJ 1.48v; National Institutes of Health, USA). From the three obtained images only the image which showed the least pressure application was selected and digitized on three consecutive days. The tendon CSA was manually outlined (excluding the paratenon) and calculated by the software (Fig. 4a). The mean value of three measurements of the same image was defined as distal or proximal CSA, respectively.

To estimate the effect of pressure on the AT CSA, two images with minimal and maximal pressure (Fig. 3a, b) were exported (VirtualDub, 1.10.4) and digitized. Analysts who determined AT CSA, AT thickness, and distance (gel pad–skin surface) were blinded.

#### MRI examinations

Magnetic resonance imaging examination took place in the MRI-laboratory of the University of Graz and Technical University Graz. A 3T scanner (Magnetom Skyra; Siemens, Erlangen, Germany) and a head coil (Head/Neck 20; Siemens) were used (T1-weighted, TR/TE 700/21; FOV 13.0 × 13.0 cm; pixel size 0.34 × 0.34 mm, slice thickness 1.1 mm, spacing between slices 0.2 mm) to obtain the images.

Before scanning, the custom-made splint was placed onto the subject’s right ankle joint to ensure the predefined position during MRI measurement. The ankle joint angle was controlled by ensuring the positions of the alignment lines between sleeve and skin (Fig. 1).

Two spherical markers (8.45 mm diameter) were carefully placed laterally (Fig. 2b) with their center at the level of the dashed lines. Thus, the MRI images including the spherical markers with the largest diameter corresponded to the US scanning position and were therefore used for further analysis.

The subject was positioned supine with knees fully extended and the ankle joint was carefully placed with small weight bags in the head coil. Sagittal images were recorded and used to adjust the FOV at a right angle to the AT path at the level of both spherical markers. Finally, 12 transversal images for each measurement position (distal, proximal) were obtained. Two out of 24 images, which contained the spherical markers with their largest diameter (Fig. 4b), were exported and the CSA was measured on three consecutive days with ImageJ as follows: At first, the images were converted (32 bit grayscale) and calibrated.

Subsequently, an adjustable threshold cut-off method was used to identify the AT boundaries (Fig. 4b). The selection of the appropriate threshold cut-off was standardized for both examiners by the following criterion: The threshold was adjusted until the smallest CSA representing the natural appearance of the AT CSA (nearly oval shape with round boundaries) was visible. The outlined CSA was further calculated automatically by the software and the mean value of three measurements of the same image was defined as CSA.

The accuracy of the threshold cut-off method was validated by measuring the CSA of the spherical markers: The diameter of the capsule used as marker was measured with a micro caliper to the nearest 0.05 mm. The measured diameter was 8.45 mm which corresponds to a calculated maximal marker CSA of 56.1 mm^2^. This value was compared to the mean marker area measured in eight different MRI images using the threshold cut-off method.

### Statistical analyses

All statistical analyses were performed with SPSS (version 22.0, SPSS Inc, Chicago, IL, USA). The level of significance was set to *α* = 0.05.

#### US-MRI comparison

At first, an independent *t* test was performed to compare the MRI image analysis of both raters. As a result, the collapsed MRI data (rater 1 + rater 2) was used for further analysis. Furthermore, as a result of the ANOVA analysis (see below), the collapsed US data was used for the comparison with the collapsed MRI data.

A mixed within-between two-way repeated measures ANOVA (independent variables: within = method (US-MRI), between = raters) was used to compare US and MRI findings.

#### US and MRI reliability

For reliability analysis of US and MRI measurements, coefficients of variation (CV) and intraclass correlation coefficients [ICC_(2,2)_ (95% CI)] were used. Furthermore, standard error of measurement (SEM) and minimal detectable change (MDC_95_) with a confidence level of 95% were calculated. SEM as an indicator of absolute reliability (i.e., degree to which repeated measurements vary for individuals; Atkinson and Nevill 1998) was determined by the following formula (Atkinson and Nevill 1998; Hars et al. 2013): ${\text{SEM}} = {\text{SD}} \times \sqrt 1 - {\text{ICC}}$. Subsequently, the SEM was used to calculate the MDC_95_ as a measure of sensitivity to change (Hars et al. 2013): $1.96 \times \sqrt 2 \times {\text{SEM}}$.

To assess the effect between raters and US sessions a comparison of the AT CSA was conducted with a mixed within-between two-way repeated measures ANOVA (independent variables: within = time, between = rater) for both (distal, proximal) positions.

#### Measurement conditions and threshold cut-off method

A paired *t* test was performed to investigate the effect of the applied probe pressure on the AT CSA, thickness, and distance (gel pad–skin surface).

Validation of the threshold cut-off method was conducted by use of a one-sample *t* test.

## Results

### US-MRI comparison

A significant main method effect (US/MRI) was found for both the distal (Wilk’s lambda = 0.59, *F* (1, 28) = 19.46, *p* < 0.001, *η* ^2^ = 0.41) and proximal (Wilk’s lambda = 0.47, *F* (1, 28) = 31.48, *p* < 0.001, *η* ^2^ = 0.53) position. Mean US values are listed in Table 2, mean MRI CSA values in Table 3.

**Table 2:** Summary of US AT CSA measurements (mean ± SD) showing coefficients of variation (CV), intraclass correlation coefficients (ICC_(2,2)_), standard error of measurement (SEM), minimal detectable change (MDC_95_), confidence interval (95% CI) for intra- and inter-rater reliability for raters (1, 2), measurement positions (distal, proximal) and sessions (1 and 2)

	Distal				ICC_(2,2)_	95% CI	SEM	MDC_95_	Proximal				ICC_(2,2)_	95% CI	SEM	MDC_95_
	Session 1		Session 2						Session 1		Session 2					
	AT CSA	CV	AT CSA	CV					AT CSA	CV	AT CSA	CV				
US																
Intra-rater																
Rater 1	56.5 ± 10.8	4.7	54.9 ± 8.8	3.7	0.93	0.78–0.96	0.77	2.16	49.7 ± 7.7	3.6	49.4 ± 6.3	4.9	0.89	0.67–0.96	0.70	1.94
Rater 2	60.2 ± 6.3	1.9	60.6 ± 8.3	1.5	0.90	0.71–0.97	0.73	2.05	54.4 ± 7.5	1.6	55.1 ± 7.7	1.7	0.97	0.91–0.99	0.22	0.61
Inter-rater	58.1 ± 8.2	7.3	–	–	0.89	0.01–0.97	1.22	3.38	52.2 ± 6.9	7.1	–	–	0.84	0.16–0.97	1.47	4.07

AT-CSA in mm^2^; CV in %; SEM in mm^2^; MDC_95_ in mm^2^

**Table 3:** Summary of MRI AT CSA measurements (mean ± SD) showing coefficients of variation (CV), intraclass correlation coefficients (ICC_(2,2)_), standard error of measurement (SEM), minimal detectable change (MDC_95_), confidence interval (95% CI) for intra- and inter-rater reliability for raters (1, 2) and measurement positions (distal, proximal)

	AT CSA	Distal					AT CSA	Proximal				
		CV	ICC_(2,2)_	95% CI	SEM	MDC_95_		CV	ICC_(2,2)_	95% CI	SEM	MDC_95_
MRI												
Intra-rater												
Rater 1	59.1 ± 8.5	3.7	–	–	–	–	54.4 ± 8.7	3.4	–	–	–	–
Rater 2	62.7 ± 7.9	1.5	–	–	–	–	57.1 ± 8.0	1.0	–	–	–	–
Inter-rater	60.9 ± 8.2	4.6	0.94	0.04–0.99	0.63	1.76	55.7 ± 8.3	3.9	0.97	0.16–0.99	0.34	0.94

AT-CSA in mm^2^; CV in %; SEM in mm^2^; MDC_95_ in mm^2^

The US method used for image analysis underestimated the CSA by ~4.6% (collapsed data: US/MRI 58.1 ± 8.6/60.9 ± 8.3 mm^2^) and ~6.3% (collapsed data: US/MRI 52.2 ± 7.4/55.7 ± 8.3 mm^2^) for the distal and proximal position, respectively (average ~5.5%).

We refrained from conducting Bland–Altman analyses since the mean differences of both methods (US, MRI) controlled with a one sample *t* test, a precondition for Bland–Altman analysis, already showed a systematically significant difference (*p* < 0.001) for both measurement positions (distal, proximal).

### US and MRI reliability

The ANOVA analysis between raters and sessions for the US measurements showed no significant main (RATER and TIME) or interaction effect for both the distal (Wilk’s lambda = 0.959, *F* (1, 28) = 1.2, *p* > 0.05, effect size = 0.04) and proximal (Wilk’s lambda = 0.973, *F* (1, 28) = 0.8, *p* > 0.05, effect size = 0.027) measurement position. As a consequence, the collapsed US data were used for US and MRI comparison (see also “Statistical analyses”).

The mean CVs of US measurements ranged from 1.5 to 4.7% and 1.6 to 4.9% for the distal and proximal positions, respectively (Table 2). ICC values showed excellent intra-rater reliability for the distal (Table 2) and good to excellent reliability for the proximal position (Portney and Watkins 2008). SEM and MDC_95_ values (Table 2) ranged from 0.22 to 0.77 mm^2^ and 0.61 to 2.16 mm^2^, respectively. Inter-rater reliability analysis revealed good ICC values for both positions. SEM and MDC_95_ values for the distal and proximal position were 1.22 and 3.38 mm^2^ as well as 1.47 and 4.07 mm^2^, respectively (Table 2).

The mean CVs of MRI measurements ranged from 1.5 to 3.7% and 1.0 to 3.4% for the distal and proximal positions, respectively (Table 3). The ICC values for the inter-rater analysis were excellent for the distal and proximal position and we found low SEM (0.34–0.63 mm^2^) and MDC_95_ (0.94–1.76 mm^2^) values in both positions (Table 3). Moreover, no significant (*p* > 0.05) differences were found both between raters and positions.

### Measurement conditions and threshold cut-off method

**Fig. 5:**
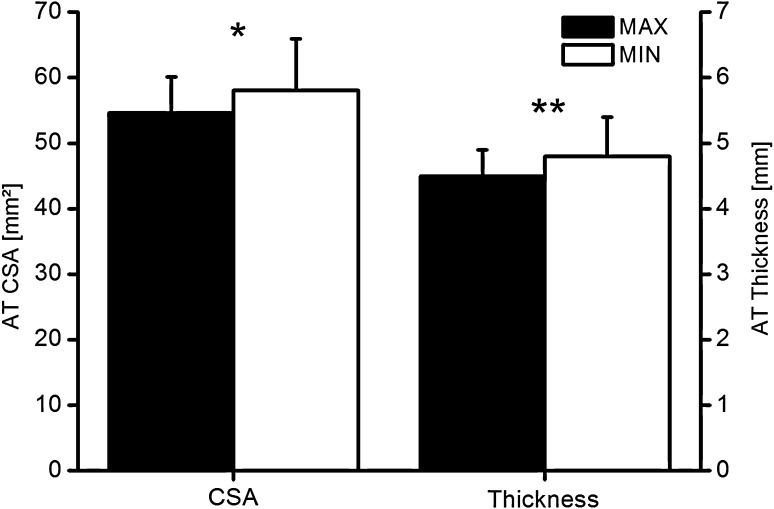
Mean (±SD) of the Achilles tendon CSA and thickness due to minimal and maximal pressure applied from the US probe on the AT. *AT* Achilles tendon, *CSA* cross-sectional area; *significant difference (*p* < 0.05); **significant difference (*p* < 0.001)

Probe pressure (Fig. 5) significantly affected (*p* < 0.05) AT CSA (max/min 54.6 ± 5.5/58.1 ± 7.8 mm^2^), tendon thickness (max/min 4.5 ± 0.4/4.8 ± 0.6 mm), and (*p* < 0.001) the distance (max/min 1.1 ± 0.5/12.1 ± 2.1 mm).

For the validation of the threshold cut-off method, the one-sample *t* test showed a statistically significant (*p* < 0.001) underestimation (~2.4 mm^2^ or ~4.2%) of the marker CSA analyzed in the images (53.7 ± 1.0 mm^2^) compared to the calculated CSA (56.1 mm^2^) of the spherical marker.

## Discussion

The major finding of this study was that US delivered systematically smaller AT CSA values when compared to MRI. Therefore, both methods cannot be used interchangeably for the investigation of the AT area. However, US and MRI separately showed good reliability.

### US-MRI comparison

Several explanations can be attributed to the observed difference between US and MRI findings.

Owing to considerable variability between tendon courses and the imaging planes (Kartus et al. 2000), obtaining comparable images of tendon tissue with US crucially depends on the equality of the measurement locations. A previous study (Brushoj et al. 2006) demonstrated that tendon dimensions differ between US and MRI. The authors attempted to use a standardized protocol with focus on the side of scanning, angulation of the transducer, and definition of the AT borders, however, no emphasis was laid on the equality of the measurement locations. They concluded that the differences in AT CSA between the two modalities may be a consequence of the different measurement locations that resulted from the variability of the CSA along tendon length (Arampatzis et al. 2010; Magnusson and Kjaer 2003). In this study, we attempted to clearly define the measurement positions by use of several mechanisms. At first, we controlled the ankle joint position by use of a splint. Comparison of the ankle joint angles showed no significant difference (*p* > 0.05) between the US measurements (session 1/2: 92.1 ± 2.2°/91.1 ± 2.1°). The same fixation procedure was also applied during the MRI measurements. Therefore, we conclude that the subject’s ankle joint angle did not differ between the measurements conducted with the different modalities. In addition, specific markers (tape, spherical markers) were used to define the corresponding measurement positions (distal, proximal) during both US and MRI measurements. Due to this standardization procedure, we are confident that the measurement positions have been identical.

In this context, another explanation for the differences between US and MRI could be transducer pressure applied during US measurements. Previous studies indicated that different degrees of transducer pressure could affect the morphological properties of the AT (Brushoj et al. 2006; Dudley-Javoroski et al. 2010; Milgrom et al. 2014). Milgrom and colleagues (2014) suggested that calculations of the AT hypertrophy are better performed by changes in the CSA rather than thickness. Our results showed that maximal probe pressure could alter (~6%) both AT CSA and thickness. Therefore, we cannot support the suggestions stated by Milgrom et al. (2014).

Additionally, the index used for pressure analysis (distance gel pad–skin surface) was significantly (*p* < 0.01) different between the distal (7.5 ± 2.6 mm) and proximal (5.9 ± 2.4 mm) position indicating that more pressure was applied at the proximal position. Since the thickness of the gel pad was 20 mm, which would indicate no pressure application, the aforementioned indices (distances) would imply a ~4% decrease of the actual AT CSA due to transducer pressure. Nevertheless, we suggest the use of a gel pad, since the inevitable probe pressure can be immediately visually controlled, while the generated error can be kept consistent throughout measurements.

### US and MRI reliability

When assessing tendon morphological and mechanical properties, it is important to know if the examined differences are related to inter-subject differences, training interventions, or influenced by measurement error. Therefore, knowledge about the precision (i.e., intra- and inter-tester reliability) of the method is crucial (Skou and Aalkjaer 2013). In the past, several researchers evaluated the use of US for the assessment of AT morphological properties (Dudley-Javoroski et al. 2010; Foure et al. 2011; Intziegianni et al. 2015; Kubo et al. 2014; Milgrom et al. 2014; Ying et al. 2003; Waugh et al. 2012).

In a recent study, Milgrom and colleagues (2014) found a high intraobserver reliability (ICC = 0.96) and a small SEM (2.6 mm^2^) for their US measurements indicating that the variation due to measurement error obtained by one observer is small when the AT CSA is assessed. Similar results (ICC = 0.99; CV = 2.2%; SEM = 0.8 mm^2^) were found by Foure et al. (2011) who examined the day-to-day reliability of their US measurements. Dudley-Javoroski et al. (2010) separated the overall variability (CV = 5.8%) of the image acquisition and analysis (tracing) of an experienced observer into its parts and reported a variability (CV = 3.83%) of the image analysis process alone. The authors further stated that the between-observer variation exceeded the within-observer variation.

It is important to note that high measurement accuracy could only be achieved when the examination procedure is well standardized. In this context, a recent study (Intziegianni et al. 2015) reported good to excellent reproducibility for the AT CSA when the assessment was conducted at 4 and 6 cm proximal to the tendon insertion (ICC of 0.86 and 0.94; SEM of 4.4 and 2.9 mm^2^, respectively). The authors also provided the limits of agreement (15.5 and 11.9 mm^2^, respectively) indicating inherent difficulties in US scanning and image analysis. Although markers (metal wires) were used in that study, the joint angle was not controlled, which could possibly have an effect on the scanning position between their measurement sessions.

In accordance with the previous studies, the present results indicate that a single rater can consistently perform US examinations of the AT CSA yielding highly reproducible results. We found lower CV, SEM, and MDC_95_ values for intra-rater reliability (average ~2.9%, 0.6, and ~1.7 mm^2^, respectively) compared to the values determined for inter-rater reliability (average 7.2%, ~1.3, and ~3.7 mm^2^, respectively).

In view of the fact that interventional studies (Arampatzis et al. 2007; Bohm et al. 2014) demonstrated possible increases of the AT CSA between 3.7 and 9.6%, the MDC_95_ value (~3% of the average AT CSA) for a single observer found in our study, may be accurate enough to detect these alterations of the AT CSA. In contrast, US inter-rater reliability showed a high MDC_95_ value (7% of the average AT CSA), which indicates that the inclusion of a second observer would decrease the measurement accuracy of the AT CSA assessment. Therefore, we agree with the previous studies (Brushoj et al. 2006; Dudley-Javoroski et al. 2010; Intziegianni et al. 2015; Milgrom et al. 2014; O’Connor et al. 2004; Ying et al. 2003) that recommend the inclusion of a single rater for US examinations of the AT CSA.

Concerning MRI intra- and inter-rater reliability, contrasting results (CVs ranged from 1.5 to 7.5%) can be found in the literature (Brushoj et al. 2006; Kubo et al. 2002; Magnusson et al. 2001). The findings of the present study (Table 3) are in good agreement with the results reported by Kubo et al. (2002).

The low inter-rater MDC_95_ values of the tendon size (2.9 and 1.7% of the mean distal and proximal AT CSA, respectively) also indicate that the MRI method is more sensitive to detect alterations of the AT CSA compared to assessments with US if measurements are to be performed by different investigators. We assume that the low CV and MDC_95_ values for both measurement positions (distal, proximal) can be also attributed to the used threshold cut-off method, which was intended to remove the observer bias.

Based on the findings above, we suggest that US can be applied in cross-sectional studies where greater differences may occur (Pang and Ying 2006; Tweedell et al. 2016). In this context, MRI could be used in prospective study designs that aim to accurately detect smaller changes in AT CSA.

### Limitations

There are a few important limitations to our study. First, we have to note that we used two different digitization methods for US and MRI image analysis. In the past, several US studies used manual tracing (Brushoj et al. 2006; Dudley-Javoroski et al. 2010; Intziegianni et al. 2015), equation-based digitization (Milgrom et al. 2014), or assumptions of the tendon shape (Kallinen and Suominen 1994) to assess the AT CSA. Moreover, MRI images were traced manually (Brushoj et al. 2006) or automatically (Hansen et al. 2003). We used manual contour tracing for US image analysis and decided to utilize an automatic tracing method for MRI image analysis. We are aware of the fact that this decision could have had an influence on the study outcomes. In this context, we first validated the used threshold cut-off method, whereby a significant underestimation of the CSA in the images (~2.4 mm^2^ or ~4.2%) compared to the measured marker was found. However, this finding does not influence the main outcome of this study since the difference between the methods would be even greater. Second, we additionally digitized the MRI images manually with the same procedure (manual contour tracing) used for US image analysis (unpublished data). This analysis also delivered a systematic difference (3.3 mm^2^) for the proximal position and differences [Bias ± LoA: 1.9 (−7.1, +10.9) mm^2^] representing 12 and 19% of the AT CSA for the distal position which exceed the expected CSA increases due to interventions. Therefore, we conclude that the differences found in this study cannot be attributed to the different digitization methods used.

In this context, another important aspect that has to be considered is the question if US really underestimated the AT CSA or if the area was overestimated with MRI. We attempted to not to include the paratenon when outlining the CSA in US images, however, we cannot preclude for certain that the paratenon is clearly identifiable when the T1-weighted MRI setting is used, and therefore, may be included in the area outlined in MRI images (Bohm et al. 2016). Although it appears that MRI underestimates the true CSA of the tendon (Couppé et al. 2014), different digitization methods could reduce that underestimation to at least 2.8% (Couppé et al. 2014). In this context, we digitized the US images with inclusion of the paratenon (Pierre-Jerome et al. 2010) and compared the AT CSA to that determined in MRI images. The US values that included the paratenon (US_PT_) showed a significant (*p* < 0.01) greater AT CSA for both the distal (MRI 62.7 ± 7.9 mm^2^; US_PT_ 78.7 ± 9.2 mm^2^) and proximal (MRI 57.1 ± 8.0 mm^2^; US_PT_ 71.3 ± 10.5 mm^2^) position. This finding indicates that by use of the T1-weighted MRI sequence, it is possible to separate the paratenon from the main CSA, and therefore, to measure the mere area of the tendon. Similar differences are reported in a previous study (Stecco et al. 2014) where the inclusion of the paratenon increased the CSA by ~40%. In our study, the increase was ~20%. This difference between studies may be explained by the different cohort and scanning positions.

Furthermore, we examined the intra- and inter-rater reliability of the MRI image analysis (tracing) procedure and did not investigate the acquisition reliability of MRI in a test–retest design (we conducted only one MRI examination). In the literature, CVs of 5.8% (Magnusson et al. 2001) and 4.5–7.5% (Hansen et al. 2003) for a test–retest design can be found which also include variations of both the acquisition and the analyzing (tracing) process. It is arguable that the differences between US and MRI modalities could have been smaller if the test–retest design had been used for the comparison, but we assume that it would not have changed the main outcome of our study. Future research is needed to clarify this issue.

Finally, we have to note that transducer pressure and the resulting alterations of the AT CSA were presented as a linear relationship in this study. However, it is known that biological structures exhibit a curvilinear force–deformation relationship with large deformations occurring in their toe region. This aspect could not be investigated due to technical limitations. Further studies are needed to examine that aspect.

## Conclusions

Our findings demonstrated that US and MRI could not be used interchangeably for the assessment of the Achilles tendon CSA, however, both methods separately showed high intra-rater reliability.

## Abbreviations

AT-CSA: Achilles tendon cross-sectional area
CV: Coefficient of variation
MDC: Minimal detectable change
MRI: Magnetic resonance imaging
SEM: Standard error of measurement
US: Ultrasound

## References

[CR1] Arampatzis A, de Monte G, Karamanidis K, Morey-Klapsing G, Stafilidis S, Bruggemann G-P (2006). Influence of the muscle-tendon unit’s mechanical and morphological properties on running economy. J Exp Biol.

[CR2] Arampatzis A, Karamanidis K, Albracht K (2007). Adaptational responses of the human Achilles tendon by modulation of the applied cyclic strain magnitude. J Exp Biol.

[CR3] Arampatzis A, Peper A, Bierbaum S, Albracht K (2010). Plasticity of human Achilles tendon mechanical and morphological properties in response to cyclic strain. J Biomech.

[CR4] Atkinson G, Nevill AM (1998). Statistical methods for assessing measurement error (reliability) in variables relevant to sports medicine. Sports Med.

[CR5] Bohm S, Mersmann F, Tettke M, Kraft M, Arampatzis A (2014). Human Achilles tendon plasticity in response to cyclic strain: effect of rate and duration. J Exp Biol.

[CR6] Bohm S, Mersmann F, Schroll A, Mäkitalo N, Arampatzis A (2016). Insufficient accuracy of the ultrasound-based determination of Achilles tendon cross-sectional area. J Biomech.

[CR7] Brushoj C, Henriksen BM, Albrecht-Beste E, Holmich P, Larsen K, Bachmann Nielsen M (2006). Reproducibility of ultrasound and magnetic resonance imaging measurements of tendon size. Acta Radiol.

[CR8] Couppé C, Svensson RB, Sødring-Elbrønd V, Hansen P, Kjaer M, Magnusson SP (2014). Accuracy of MRI technique in measuring tendon cross-sectional area. Clin Physiol Funct Imaging.

[CR9] Dudley-Javoroski S, McMullen T, Borgwardt MR, Peranich LM, Shields RK (2010). Reliability and responsiveness of musculoskeletal ultrasound in subjects with and without spinal cord injury. Ultrasound Med Biol.

[CR10] Faul F, Erdfelder E, Lang A-G, Buchner A (2007). G*Power 3: a flexible statistical power analysis program for the social, behavioral, and biomedical sciences. Behav Res Methods.

[CR11] Foure A, Nordez A, McNair P, Cornu C (2011). Effects of plyometric training on both active and passive parts of the plantarflexors series elastic component stiffness of muscle-tendon complex. Eur J Appl Physiol.

[CR12] Hansen P, Aagaard P, Kjaer M, Larsson B, Magnusson SP (2003). Effect of habitual running on human Achilles tendon load-deformation properties and cross-sectional area. J Appl Physiol.

[CR13] Hars M, Herrmann FR, Trombetti A (2013). Reliability and minimal detectable change of gait variables in community-dwelling and hospitalized older fallers. Gait Posture.

[CR14] Intziegianni K, Cassel M, König N, Müller S, Fröhlich K, Mayer F (2015). Ultrasonography for the assessment of the structural properties of the Achilles tendon in asymptomatic individuals. An intra-rater reproducibility study. Isokinet Exerc Sci.

[CR15] Jacobson JA (2005). Musculoskeletal ultrasound and MRI: which do I choose?. Semin Musculoskelet Radiol.

[CR16] Kallinen M, Suominen H (1994). Ultrasonographic measurements of the Achilles tendon in elderly athletes and sedentary men. Acta Radiol.

[CR17] Kartus J, Rostgard-Christensen L, Movin T, Lindahl S, Ejerhed L, Karlsson J (2000). Evaluation of harvested and normal patellar tendons: a reliability analyses of magnetic resonance imaging and ultrasonography. Knee Surg Sports Traumatol Arthrosc.

[CR18] Kongsgaard M, Aagaard P, Kjaer M, Magnusson SP (2005). Structural Achilles tendon properties in athletes subjected to different exercise modes and in Achilles tendon rupture patients. J Appl Physiol.

[CR19] Kubo K, Kanehisa H, Fukunaga T (2002). Effects of resistance and stretching training programmes on the viscoelastic properties of human tendon structures in vivo. J Physiol.

[CR20] Kubo K, Teshima T, Hirose N, Tsunoda N (2014). A cross-sectional study of the plantar flexor muscle and tendon during growth. Int J Sports Med.

[CR21] Magnusson SP, Kjaer M (2003). Region-specific differences in Achilles tendon cross-sectional area in runners and non-runners. Eur J Appl Physiol.

[CR22] Magnusson SP, Aagaard P, Rosager S, Dyhre-Poulsen P, Kjaer M (2001). Load-displacement properties of the human triceps surae aponeurosis in vivo. J Physiol.

[CR23] Milgrom Y, Milgrom C, Altaras T, Globus O, Zeltzer E, Finestone AS (2014). Achilles tendons hypertrophy in response to high loading training. Foot Ankle Int.

[CR24] O’Connor P, Grainger A, Morgan SR, Smith KL, Waterton JC, Nash AFP (2004). Ultrasound assessment of tendons in asymptomatic volunteers: a study of reproducibility. Eur Radiol.

[CR25] Pang BS, Ying M (2006). Sonographic measurement of the Achilles tendons in asymptomatic subjects: variation with age, body height, and dominance of ankle. J Ultrasound Med.

[CR26] Pierre-Jerome C, Moncayo V, Terk MR (2010). MRI of the Achilles tendon: a comprehensive review of the anatomy, biomechanics, and imaging of overuse tendinopathies. Acta Radiol.

[CR27] Portney LG, Watkins MP (2008). Foundations of clinical research: applications to practice.

[CR28] Rasmusson OS (2000). Sonography of tendons. Scand J Med Sci Sports.

[CR29] Rosager S, Aagaard P, Dyhre-Poulsen P, Neergaard K, Kjaer M, Magnusson SP (2002). Load-displacement properties of the human triceps surae aponeurosis and tendon in runners and non-runners. Scand J Med Sci Sports.

[CR30] Skou ST, Aalkjaer JM (2013). Ultrasonographic measurement of patellar tendon thickness–a study of intra- and interobserver reliability. Clin Imaging.

[CR31] Stecco C, Cappellari A, Macchi V, Porzionato A, Morra A, Berizzi A, De Caro R (2014). The paratendineous tissues: an anatomical study of their role in pathogenesis of tendinopathy. Surg Radiol Anat.

[CR32] Tweedell AJ, Ryan ED, Scharville MJ, Rosenberg JG, Sobolewski EJ, Kleinberg CR (2016). The influence of ultrasound measurement techniques on the age-related differences in Achilles tendon size. Exp Gerontol.

[CR33] Waugh CM, Blazevich AJ, Fath F, Korff T (2012). Age-related changes in mechanical properties of the Achilles tendon. J Anat.

[CR34] Ying M, Yeung E, Li B, Li W, Lui M, Tsoi C-W (2003). Sonographic evaluation of the size of Achilles tendon: the effect of exercise and dominance of the ankle. Ultrasound Med Biol.

